# Structural brain differences in professional Australian rules footballers following mild traumatic brain injury: When head size matters

**DOI:** 10.3389/fneur.2026.1701097

**Published:** 2026-01-16

**Authors:** Jackson M. Lee, Heath R. Pardoe, Donna M. Parker, Mangor Pedersen, Michael Makdissi, David F. Abbott, Graeme D. Jackson, Remika Mito

**Affiliations:** 1 The Florey Institute of Neuroscience and Mental Health, Melbourne, VIC, Australia; 2 Department of Psychiatry, The University of Melbourne, Melbourne, VIC, Australia; 3 Florey Department of Neuroscience and Mental Health, The University of Melbourne, Melbourne, VIC, Australia; 4 Department of Psychology and Neuroscience, Auckland University of Technology, Auckland, New Zealand; 5 LaTrobe Sport and Exercise Medicine Research Centre, La Trobe University, Melbourne, VIC, Australia; 6 Australian Football League, Melbourne, VIC, Australia; 7 Department of Neurology, Austin Health, Melbourne, VIC, Australia

**Keywords:** hippocampus, intracranial volume, MRI, neuroimaging, sports-related concussion

## Abstract

**Introduction:**

Concussion, a type of mild traumatic brain injury common in collision sports, is thought to be associated with subtle brain changes that are not visually appreciable on conventional neuroimaging. This study quantified differences in subcortical volumes from structural MRI between 31 recently concussed professional Australian rules footballers (within 3 months of injury) and 37 healthy, non-athlete controls.

**Methods:**

T1-weighted MRI were acquired at 3 T and processed using FreeSurfer. Hippocampal and amygdala volumes were normalized by estimated total intracranial volume (eTIV). Longitudinal changes were assessed in a subset of 12 footballers with follow-up MRI. Cortical thickness differences were also explored using vertex-wise analysis.

**Results:**

Footballers exhibited lower proportional hippocampal and amygdala volumes, and reduced cortical thickness compared to controls. However, after exploring different methodological approaches for estimating intracranial volume (ICV), volumetric findings were seen to vary based on the ICV estimation method used for normalization.

**Discussion:**

This study demonstrates subtle, likely persistent neuroanatomical differences between professional Australian rules footballers and non-athlete controls. Importantly, we advocate for cautious clinical interpretation of volumetric MRI findings considering methodological variabilities, particularly when inherent cohort differences (such as ICV) may bias results, and provide recommendations for future studies that examine volumetric changes in concussion cohorts.

## 1 Introduction

A concussion is a type of mild traumatic brain injury (mTBI) characterized by a complex neurometabolic cascade of events (1) and substantial heterogeneity in clinical outcomes (2). Following a concussion, individuals may experience a range of physical, cognitive, and emotional symptoms (3) that typically resolve within days or weeks following injury; however, in some individuals these symptoms persist (3, 4). Over the past two decades, concussion has transformed from a relatively underappreciated injury to a growing public health concern, largely driven by ongoing discussion surrounding both acute and chronic neuropsychiatric sequelae (5), and the risk of potential long-term neurodegenerative consequences following repeated head impacts (6–9). Notably, individuals with a history of mTBI exhibit a substantially increased long-term risk of depression that persists years beyond the initial injury (10). Based on clinical symptoms alone, these outcomes are difficult to predict, prompting the need for acute biological markers.

Magnetic resonance imaging (MRI) provides a non-invasive method of exploring human brain structure and function *in vivo*. MRI-based biomarkers are becoming increasingly valuable in neurology (11). Structural MRI is commonly used in clinical practice following brain trauma, as potentially life-threatening brain injuries can be visually appreciated (12). However, mTBI is explicitly defined by the “absence of macroscopic neural damage” (1), meaning that structural abnormalities are not visible upon qualitative inspection of MRI (3, 13, 14). Advancements in quantitative post-processing and analysis methods have enabled the identification of neuroanatomical differences from standard structural MRI following concussion, which may assist in detecting the more subtle effects of injury (13, 15). Quantitative assessment of regional brain volumes and cortical thickness may allow for the detection of subtle group-level differences that are not visually apparent.

Previous research suggests that subcortical structures, particularly the hippocampus and amygdala, may be vulnerable to concussion (14). In the sports-related concussion literature, such findings have been observed in both athletes who have sustained a recent concussion (16), and retired athletes with a concussion history (17, 18).

Australian rules football is a full-contact sport, and is one of Australia’s most popular sporting codes (19). In contrast to contact sports such as American football and ice hockey, Australian rules football features distinct rules and playing styles, with no requirement for protective equipment (19, 20). In the professional male league, the most recently reported concussion incidence is 6.66 per 1,000 player hours (21), while some studies have documented rates of up to 17.6 per 1,000 player hours across different levels (ranging from community to elite leagues) (22, 23).

In previous work, we have identified microstructural and functional brain changes in professional Australian rules footballers using advanced imaging measures (24–26). However, it remains unclear whether subtle macrostructural differences in regional brain volume and cortical thickness are present in this cohort. Indeed, as structural MRI is more routinely obtained in individuals following concussion than more complex diffusion and functional imaging, the identification of important injury-related changes could make for valuable biomarkers.

Here, we examined subtle structural brain differences in a cohort of 31 professional Australian rules footballers with sports-related mTBI compared to healthy, non-athlete controls. Based on previous literature, we focused particularly on differences in hippocampal and amygdala volumes, while also exploring differences in cortical thickness between cohorts. We considered the potential effect of time since injury both cross-sectionally (by dividing the concussion cohort into acute and sub-acute groups), and longitudinally (in a subset of participants with follow-up MRI scans). It was hypothesized that recently concussed footballers would exhibit volumetric differences in the hippocampus and amygdala compared to controls, regardless of time since the injury. Finally, given the numerous choices available for estimation, we also explored the effect of two additional methods for intracranial volume estimation on our volumetric findings.

## 2 Materials and methods

### 2.1 Participants

Participants were selected from a retrospective dataset of professional male Australian rules footballers from the Australian Football League (AFL), who were recruited into a neuroimaging study (between 2010 and 2019) after suffering a suspected concussion (*n* = 35). All recruited players had concussion symptoms assessed by experienced club doctors who take a consistent approach to mTBI diagnosis and management (27). Players were assessed using the Sports Concussion Assessment Tool (3rd edition; SCAT3) and had clinical signs and symptoms of mTBI lasting 72 h or longer. Players were excluded from this study if their initial concussion diagnosis was not later confirmed by a doctor (*n* = 1), if the date of injury was not known (*n* = 1), or if imaging was undertaken more than 3 months after injury (*n* = 2). In total, 31 professional Australian rules footballers were included in this study.

Given the variable time since injury, players were also categorized into either an ‘acute mTBI’ group (*n* = 17; MRI scan ≤ 12 days following concussion) or a ‘sub-acute mTBI’ group (*n* = 14; MRI scan > 12 days following concussion) for the purposes of exploratory sub-analyses. The 12-day cut-off was selected in line with our previous work examining white matter changes in the same cohort (24) and the most recent AFL concussion protocol, which mandates the earliest return-to-play at 12 days following concussion (28). In addition, a subset of players were re-recruited into the study, either after they had suffered another concussion or had recovered from injury. Where players were re-recruited after another suspected mTBI, the earliest reported concussion was included to minimize the likelihood of known prior concussions influencing potential neuroanatomical findings. Players who were re-recruited after they had recovered from injury (follow-up scan, *n* = 12) were explored longitudinally.

Healthy male, non-athlete controls (*n* = 38), who were matched in age were included in this study. Control participants had no history of head trauma or concussion, and were otherwise deemed neurologically healthy. One control participant was omitted from the study due to errors in processing during structural analysis. In total, 37 control participants were included for further analysis.

All participants provided written informed consent before participating in the study. The study was approved by the human research ethics committees at the University of Melbourne (ID: 0830367) and Austin Health (ID: 49573/2019 and H2012/04475).

### 2.2 MRI data acquisition

All participants had isotropic T1-weighted images obtained using a magnetization-prepared acquisition gradient echo (MPRAGE) sequence, which was acquired on one of two 3 T Siemens scanners (Erlangen, Germany) with the following parameters: (1) Siemens Skyra (*n* = 56; healthy controls: 29): acquisition matrix of 256 × 256 × 192, voxel size = 0.9 mm^3^, inversion time = 900 ms, flip angle = 9°, TR/TE = 1900/2.5 ms. (2) Siemens Trio (*n* = 12; healthy controls: 8): acquisition matrix of 256 × 256 × 192, voxel size = 0.9 mm^3^, inversion time = 900 ms, flip angle = 9°, TR/TE = 1900/2.6 ms. Each scan underwent routine inspection by a neurologist to rule out any structural abnormalities, and were visually assessed for artifacts by the first author (J.L.) using an in-house quality assurance rating tool (29). All scans were deemed satisfactory for further analysis.

### 2.3 Structural MRI processing

Cross-sectional structural processing was performed using the *recon-all* pipeline available from FreeSurfer (v7.3.2) (30). Each subject’s hippocampal and amygdala volumes and estimated total intracranial volume (eTIV) (31) were extracted using the FreeSurfer subcortical segmentation pipeline (30). Manual quality control was performed by the first author (J.L.), who inspected each *recon-all* output for skull-stripping and intensity normalization errors. Scans were reviewed in all planes, and confirmed errors were corrected in FreeView using recon-edit, followed by reprocessing and final review. Hippocampal and amygdala volumes were normalized by eTIV using the proportion approach (32, 33). Details on this approach and its justification are provided in the Supplementary material.

### 2.4 Longitudinal analysis

Longitudinal changes in proportional hippocampal and amygdala volume were explored in 12 Australian rules footballers who had follow-up scans available (acute mTBI: *n* = 5; sub-acute mTBI: *n* = 7), using FreeSurfer’s longitudinal processing pipeline (34). Each participant had two scans: one following their earliest diagnosed concussion (timepoint 1), and a follow-up scan after recovery (timepoint 2). All follow-up scans were obtained when players were deemed functionally recovered from the concussion event.

### 2.5 Cognitive data

Cognitive screening was performed using the CogSport computerized cognitive screening battery (CogState Ltd., Melbourne, VIC, Australia) on the same day as neuroimaging. Normalized one-back and continuous learning scores, benchmarked to neurologically healthy individuals (mean = 100), were used in subsequent correlation analyses to examine the relationship between hippocampal volume and cognitive function post-mTBI. Data were unavailable for six participants, leaving 25 for analysis.

### 2.6 Exploratory intracranial volume comparison

To explore how the choice of ICV estimation method influenced volumetric findings, additional exploratory analyses were performed using sbTIV (segmentation-based total intracranial volume) (35, 36), and supratentorial volume as ICV estimates. Details on each estimation method are provided in the Supplementary material.

### 2.7 Statistical analysis

Statistical analyses of ROI volumes were performed in R (version 4.2.2) (37). Normality assumptions were tested using Levene’s and Shapiro–Wilk tests alongside visual inspection of normal probability plots (Q-Q plots). For all tests, results at *p* < 0.05 were deemed statistically significant. Given the likely non-independence of the ROI measures, we report here uncorrected *p*-values at a statistical significance threshold of *p* < 0.05. For transparency, we also report significance at a Bonferroni-corrected threshold for the four ROI comparisons (*p* < 0.0125). Independent *t*-tests compared age, eTIV, and normalized ROI volumes between Australian rules footballers and controls. Given that our cohort included participants scanned on two MRI scanners (albeit with optimally matched protocols), we additionally performed sensitivity analyses to examine the impact of scanner on our primary findings, for which results are presented in the Supplementary Tables 1–3. For exploratory analysis where the mTBI cohort was stratified based on days since injury, one-way ANOVAs were used to compare group differences age, eTIV, and normalized ROI volumes. The mTBI sub-groups were also compared based on number of AFL games played using an independent *t*-test, with post-hoc Tukey HSD tests for significant effects. Effect sizes were estimated using Cohen’s *d* for statistically significant group effects. For transparency, absolute ROI volumes for controls and footballers are provided in Supplementary Table 4. Paired *t*-tests were used to explore longitudinal ROI volume changes between timepoints. Exploratory post-hoc correlation analyses between hippocampal volume and CogSport scores were performed using Pearson correlation coefficients. Cortical thickness was analyzed using vertex-wise general linear models in FreeSurfer, with maps co-registered to the *fsaverage* template, smoothed (10 mm full-width half-maximum Gaussian filter), and age (demeaned) included as a covariate. Family-wise error was controlled using Monte Carlo simulation (cluster-forming threshold *p* < 0.001) (38), with Bonferroni correction applied across hemispheres, and results deemed significant at a corrected cluster-wise threshold of *p* < 0.05. The same analysis was repeated once the mTBI cohort was stratified based on time since injury. As part of the exploratory ICV comparison analysis, each ROI volume was also normalized using both supratentorial volume and sbTIV. Independent *t*-tests assessed group differences in the ROI volumes normalized with each ICV method. To evaluate agreement between ICV estimates, Bland–Altman plots were created, whereby the differences between ICV estimates (Δ ICV) were plotted against mean of the two ICV estimates (i.e., (ICV estimate_1_ + ICV estimate_2_) ÷ 2) (39). For each plot, 95% agreement intervals were used as calculated by ΔICV ±1.96 standard deviations. To quantitatively interpret the bias between methods, Pearson correlation coefficients were calculated between ΔICV and mean ICV (40). A stronger, significant correlation would indicate a greater proportional bias between ICV methods; in other words, the agreement between two ICV estimates would significantly vary as ICV estimates change. Pearson correlation coefficients were also calculated for all pairwise comparisons of ICV estimates.

## 3 Results

### 3.1 Demographics

Participant demographics are summarised in Table 1. Australian rules footballers exhibited significantly greater eTIV compared to controls (*p* < 0.001; *t*(66) = 3.65). Once the mTBI cohort was stratified into acute and sub-acute groups, post-hoc Tukey’s HSD tests revealed significant differences in eTIV compared to controls for both the acute mTBI cohort (*p* = 0.029), and sub-acute mTBI cohort (*p* = 0.005). There was no significant difference in age between groups or AFL experience between the mTBI sub-groups.

**Table 1:** Group demographics and characteristics.

Group	Age (years)	Intracranial volume (eTIV) (cm^3^)	Time since mTBI (days)	AFL games played^a^
mTBI	24.43 (3.47)	1779.00 (141.79)	*–*	*–*
Acute	23.92 (3.43)	1761.55 (133.53)	6.88 (3.46)	80.41 (85.71)
Sub-acute	25.04 (3.54)	1800.18 (153.52)	35.49 (19.65)	63.36 (57.49)
Controls	24.91 (5.50)	1648.84 (150.18)	*–*	*–*
**Statistic, *p*-value**	*t*(66) = −0.43, *p* = 0.672 *F*(2, 65) = 0.31, *p* = 0.735	*t*(66) = 3.65, ***p* < 0.001** *F*(2, 65) = 6.88, ***p* = 0.002**	*–*	*t*(28) = 0.66, *p = 0.*515

Values are means (standard deviation). a^:^ AFL games played at time of mTBI. Significant *p*-values shown in bold. *F*-statistics compare controls and mTBI sub-groups; *t*-statistics compare controls vs. whole mTBI cohort, or acute vs. sub-acute (for AFL games).

#### 3.1.1 Proportional volumetric findings (mTBI cohort vs. controls)

**Figure 1:**
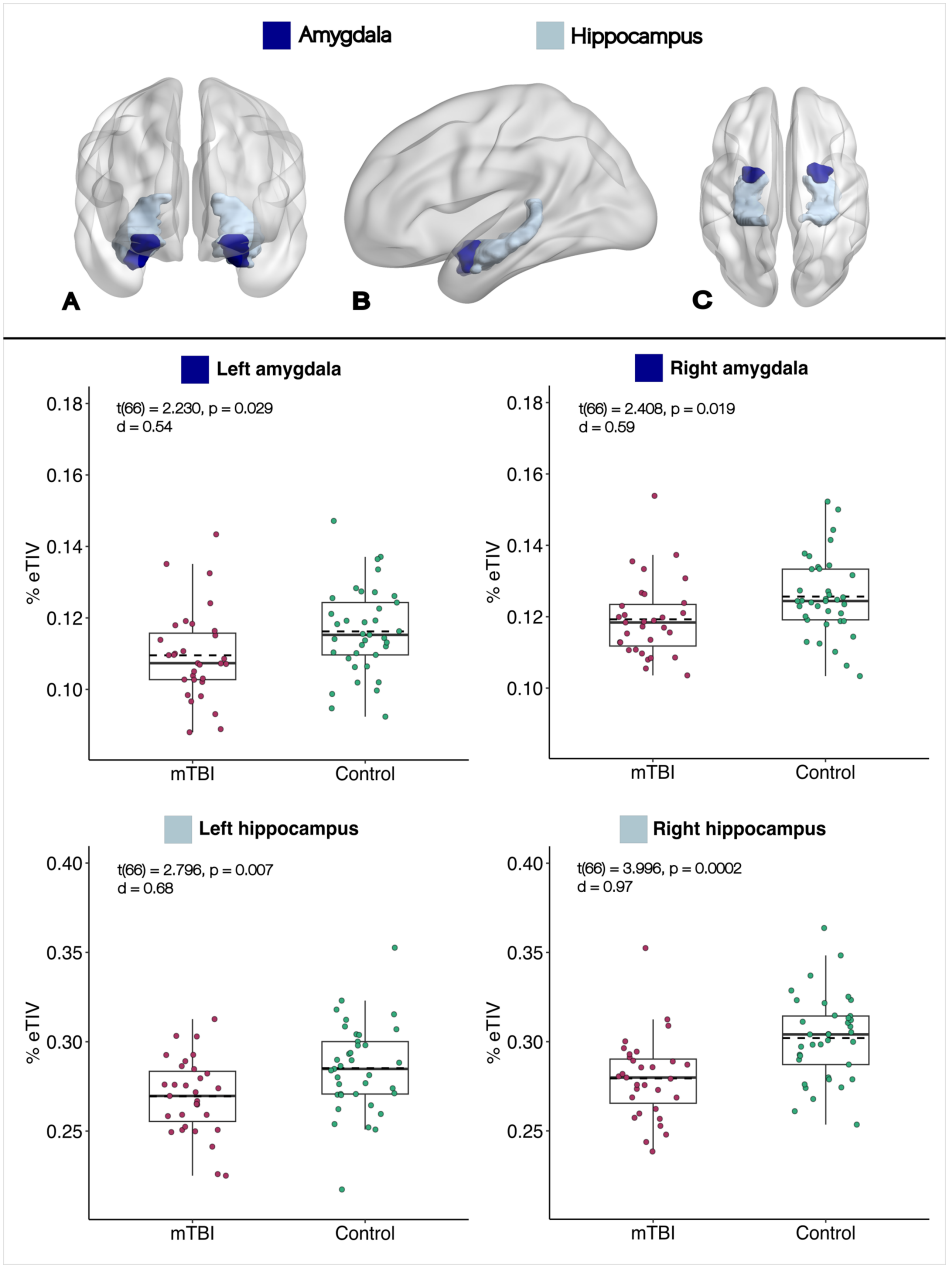
Normalized ROI volumes (mTBI and controls). Top: 3D render of hippocampus (light blue) and amygdala (dark blue) using BrainNet Viewer (75): **(A)** Coronal; **(B)** sagittal; **(C)** axial. Bottom: Box plots of normalized volumes (solid line = median; dotted line = mean; whiskers = ±1.5 × IQR). *T*-statistics, uncorrected *p*-values, and Cohen’s *d* are reported for each ROI.

Professional footballers showed significantly lower bilateral proportional hippocampal and amygdala volumes compared to controls (*p* < 0.05; see Figure 1). After Bonferroni correction (*p* < 0.0125), bilateral hippocampal differences remained significant.

#### 3.1.2 Proportional volumetric findings (mTBI sub-groups vs. controls)

Compared to controls, each mTBI sub-group displayed lower proportional right hippocampal volume [acute: *p* = 0.005; sub-acute: *p* = 0.008; *F*(2, 65) = 7.87]. The sub-acute group also exhibited lower proportional left hippocampal volumes [*p* = 0.028; *F*(2, 65) = 4.16]. Proportional right amygdala volume was lower in the acute mTBI group compared to controls [*p* = 0.045; *F* (2, 65) = 3.21]. Differences in left hippocampal and right amygdala volumes did not survive Bonferroni correction. No significant differences were observed between acute and sub-acute groups (see Supplementary Table 5 and Supplementary Figure 1).

### 3.2 Longitudinal volumetric findings

In mTBI participants with longitudinal data available (*n* = 12), no significant changes were observed in subcortical volumes across timepoints. A reduction in proportional right hippocampal volume was observed [*p* = 0.042; *t*(11) = 2.31], however this did not survive Bonferroni correction (*p* < 0.0125). All other ROIs showed no significant change (see Table 2 and Supplementary Figures 2, 3).

**Table 2:** Longitudinal changes in ROI volumes.

ROI	Mean proportional volume		Statistic	*p*-value
	Timepoint 1	Timepoint 2		
Left hippocampus	0.269	0.268	*t*(11) = 1.22	0.249
Right hippocampus	0.280	0.277	*t*(11) = 2.31	**0.042**
Left amygdala	0.111	0.110	*t*(11) = 0.86	0.409
Right amygdala	0.122	0.122	*t*(11) = 0.02	0.982

Mean proportional volumes shown for each ROI at timepoint 1 and 2. Significant uncorrected *p*-values shown in bold.

### 3.3 Cognitive correlation analyses

mTBI participants showed a significant relationship between poorer CogSport learning scores, and smaller proportional left hippocampal volume [*R* (23) = 0.44, *p* = 0.030], and right hippocampal volume [*R* (23) = 0.40, *p* = 0.046]. Although not statistically significant, there was a positive trend between mTBI participant’s CogSport one-back scores, and both left hippocampal volume [*R* (23) = 0.37, *p* = 0.069], and right hippocampal volume [*R* (23) = 0.31, *p* = 0.137] (see Supplementary Figure 4).

### 3.4 Cortical thickness findings

Vertex-wise analysis revealed a significant cluster of lower cortical thickness in the right lateral orbitofrontal cortex in recently concussed footballers compared to controls (peak *p* = 0.013; see Supplementary Figure 5). No significant group differences were observed when stratifying the mTBI cohort at a cluster-forming threshold of *p* < 0.001, and a corrected cluster-wise threshold of *p* < 0.05.

### 3.5 Exploratory ICV comparisons

**Figure 2:**
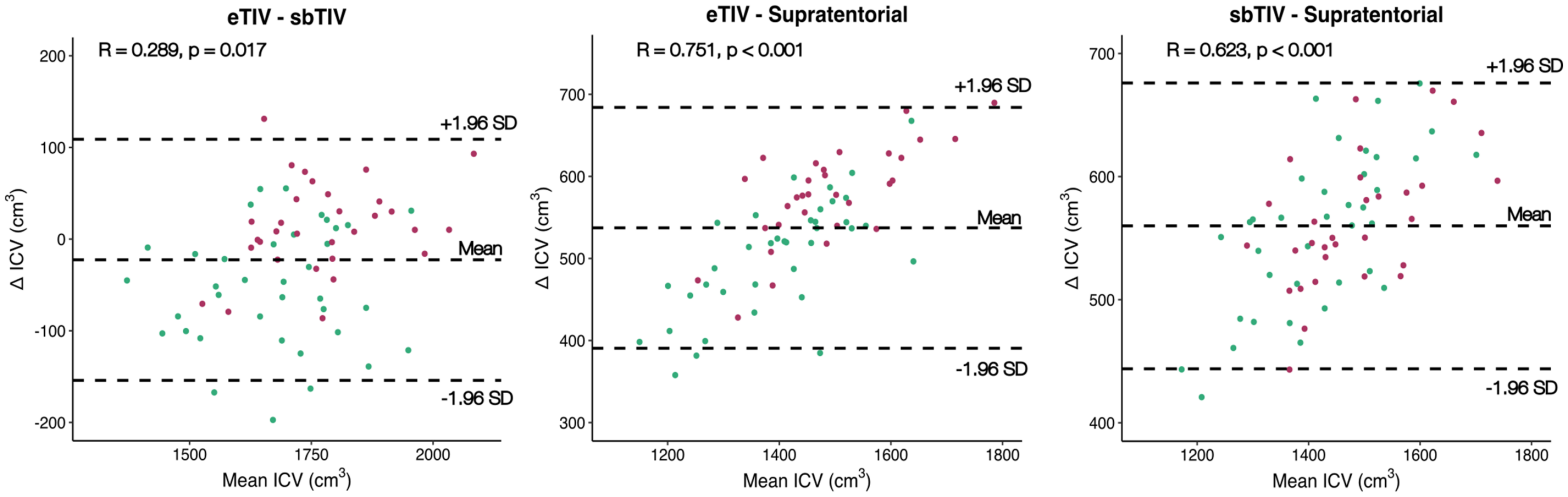
Bland–Altman comparing FreeSurfer ICV estimates. The mean ICV between methods (*x*-axis) is plotted against percentage differences (ΔICV) (*y*-axis). Pearson correlation coefficient between mean ICV and ΔICV (with *p*-value) is shown above each plot. Footballers shown as purple dots. Controls shown as green dots. Dashed lines indicate mean bias (±1.96 standard deviations) between estimates.

Three different FreeSurfer-derived ICV estimates were compared for ROI volumetric analysis. Group differences between footballers and controls were significant in eTIV [*p* < 0.001; *t*(66) = 3.65] and supratentorial volume [*p* = 0.026; *t*(66) = 2.28], but not for sbTIV. Normalising ROI volumes by sbTIV and supratentorial volume revealed a significant difference only in right hippocampal volume, unlike ROI normalisation with eTIV (all other ROIs *p* > 0.05) (see Table 3). When stratified by mTBI status, no significant group differences in proportional hippocampal or amygdala volumes (bilaterally) were observed using sbTIV or supratentorial volume for normalisation (see Supplementary Table 6). All ICV methods were strongly correlated (*R* > 0.90; see Supplementary Figure 6). However, for all ICV comparisons there was a significant proportional bias between mean ICV and ∆ICV. In other words, the agreement between ICV methods varied across different measures of ICV; specifically, greater disagreement between measures was seen at larger ICV estimates. The strongest proportional bias was seen with comparisons made to supratentorial volume [eTIV ~ supratentorial volume: *R* (66) = 0.75, *p* < 0.001, sbTIV ~ supratentorial volume: *R* (66) = 0.62, *p* < 0.001] (see Figure 2). A significant but weaker bias was observed between eTIV and sbTIV [*R* (66) = 0.29, *p* = 0.017].

**Table 3:** Group comparisons of ROI volumes normalized by different ICV estimates.

ROI	eTIV		sbTIV		Supratentorial volume	
	Statistic (and *p*-value)	Cohen’s *d*	Statistic (and *p*-value)	Cohen’s *d*	Statistic (and *p*-value)	Cohen’s *d*
Left hippocampus	*t*(66) = 2.80 ***p* = 0.007**	0.68	*t*(66) = 0.96 *p* = 0.341	0.23	*t*(66) = 1.44 *p* = 0.155	0.35
Right hippocampus	*t*(66) = 4.00 ***p* = 0.0002**	0.97	*t*(66) = 2.22 ***p* = 0.030**	0.54	*t*(66) = 2.64 ***p* = 0.010**	0.64
Left amygdala	*t*(66) = 2.23 ***p* = 0.029**	0.54	*t*(66) = 0.83 *p* = 0.413	0.20	*t*(66) = 1.22 *p* = 0.228	0.30
Right amygdala	*t*(66) = 2.41 ***p* = 0.019**	0.59	*t*(66) = 0.67 *p* = 0.506	0.16	*t*(66) = 1.27 *p* = 0.207	0.31

Shown are statistics (mTBI vs. controls) and Cohen’s *d* after normalization using eTIV, sbTIV, or supratentorial volume. Significant *p*-values in bold. Gray columns (eTIV) indicate results reported in Figure 1.

## 4 Discussion

This study applied quantitative structural neuroimaging techniques to investigate subtle neuroanatomical differences between Australian rules footballers post-mTBI and non-concussed controls. The key clinical findings observed were that recently concussed footballers displayed lower proportional hippocampal and amygdala volumes, and subtle cortical thickness differences, independent of the timing since injury. Hippocampal volumes also appeared to relate to learning scores in a subset of footballers who had cognitive data available. Notably, footballers had significantly larger ICV estimates than controls. Upon exploring different methodological approaches for ICV estimation, the statistically significant volumetric findings observed in the main arm of our study were only replicated at the right hippocampus using sbTIV or supratentorial volume estimates for normalization (though effect sizes were smaller in these cases). Significant proportional biases were noted between different methods (in other words, the agreement between ICV methods varied across different measures of ICV).

Taken together, this study suggests subtle neuroanatomical differences between Australian rules footballers and controls, particularly at the hippocampus, which may be related to exposure to sports-related concussion in line with previous studies. However, we recommend careful interpretation of such findings in the context of methodological differences between studies, and difficulties studying cohorts where inherent differences (such as ICV) may introduce bias.

### 4.1 Potential vulnerability of the hippocampus and amygdala following concussion

The amygdala-hippocampal complex is a known region of vulnerability to mTBI, likely due to its anatomical location and susceptibility to biomechanical forces (14, 41), with volumetric reductions in these structures well-documented in the sports-related concussion literature (14, 16–18). Here, our findings highlight the potential vulnerability of the hippocampus to concussion in professional Australian rules football players, and offer further insight into the possible brain changes that arise following mTBI. Additionally, this work builds upon previous observations of regional functional connectivity, T2-relaxometry, and microstructural white matter differences previously reported by our group in the same cohort (24–26).

Differences in hippocampal volumes appeared to be independent of time since injury and seemed to persist beyond the acute period. Indeed, there were no differences between acute and sub-acute mTBI cohorts once the mTBI group was stratified according to days since injury, and in the subset of participants with longitudinal data, no significant changes in bilateral hippocampal or amygdala volumes were observed between the first scan and follow-up. This may suggest that the structural differences observed cross-sectionally reflect ongoing consequences of the injury that persist beyond the acute injury phase. Indeed, the study cohort were professional athletes with years of training and participation in Australian rules football, a collision sport where head impacts causing mTBI are seemingly unavoidable (19). In this way, whether these volumetric differences are related to previous injury events, or to subtle difference in brain structure between the groups that are independent of concussion and/or head trauma must be considered. Previous literature suggests that, even in the absence of diagnosed concussions, repeated sub-concussive impacts sustained during participation in collision sport may contribute to subtle, progressive volumetric declines in specific hippocampal subfields over time (42). Notably, atrophy of medial temporal lobe structures such as the hippocampus and amygdala are considered structural hallmarks of neurodegenerative outcomes that have been associated with concussion (43, 44). Although exploratory, lower proportional hippocampal volume appeared to be associated with poorer learning scores, suggesting that these subtle neuroanatomical differences may indeed be clinically relevant. However, it is important to note that downstream effects of the observed neuroanatomical differences are difficult to assess in this cohort. To better understand the dynamic effects of concussive and sub-concussive events on structural brain measures, baseline cognitive and neuroimaging data, along with consistent longitudinal data and shorter post-injury delay periods, are essential. Moreover, longitudinal studies that follow athletes throughout their careers and beyond will be crucial for understanding whether these subtle changes to medial temporal lobe structures, such as those observed here, are indeed associated with long-term consequences.

### 4.2 Cortical thickness differences in Australian rules footballers following mTBI

Exploratory analyses revealed that Australian rules footballers exhibited lower cortical thickness at the right lateral orbitofrontal cortex compared to healthy controls. Similar findings of cortical thickness differences in sports-related mTBI cohorts have been reported in previous studies, although the specific regions affected vary (20, 45, 46). Given that this finding is based on exploratory analyses, it should be seen as hypothesis-generating, with further research required to corroborate this result and explore potential clinical implications.

### 4.3 Methodological considerations for volumetric normalization

In the main arm of this study, we normalized ROI volumes with FreeSurfer’s estimated intracranial volume (eTIV). Although not the main purpose of this study, we additionally explored the effect of two additional FreeSurfer-derived ICV estimates for ROI normalization, to validate our main finding. In contrast to statistically significant group-level differences in proportional hippocampal and amygdala volumes bilaterally following ROI normalization with eTIV, when volumes were normalized using both the sbTIV and supratentorial volume estimations we observed reduced effect sizes, and statistically significant group differences were only observed at the right hippocampus.

Of note, Bland–Altman plots revealed the agreement between ICV methods exhibited a significant proportional bias, consistent with findings from previous work (40). This bias suggests that ICV methods may not agree uniformly across the entire range of ICV estimates; specifically, the different ICV methods appeared to exhibit greater disagreement at larger ICV values. This is important to note, as across all ICV methods explored, Australian rules footballers were found to have larger ICV estimates compared to controls, with statistically significant differences noted for eTIV and supratentorial volume. Differences in ICV may be a distinct feature of athletes who compete in certain sports, particularly when compared to non-athlete control cohorts. Statistically significant differences in estimated ICV have been reported previously in case–control designed studies on rugby players (18), and amateur boxers (47). As such, we highlight the importance of cautious clinical interpretation in the context of unique clinical cohorts (such as the professional athletes described here), where features that may be characteristic to a cohort may influence subsequent analyses and interpretation. Although not explored here, even within a given approach, ICV estimations may be biased. For example, eTIV has previously been shown to be biased by total brain size (48), which may account for some variability in the agreement between ICV measurements observed here. This presents an opportunity for future research to ascertain whether a similar bias exists in supratentorial volume and sbTIV estimates. While outside the scope of this study, it would also be interesting to compare our results with estimates of ICV from other popular automated processing packages (49–51), especially considering previous studies have used one automated package for morphometric measures of interest and another for ICV estimation.

We wish to emphasize that we cannot conclude whether there is a *‘correct’* ICV estimation method to choose, nor are we advocating for using one ICV estimation method over alternatives; any chosen method or pre-processing step will come with unique practical considerations. The choice of ICV estimation method should instead be guided by factors unique to a study (52). Rather, we intend to highlight here that whichever ICV method researchers decide to adopt will create a ‘forking path’ that, when considered alongside the many methodological choices (or ‘researchers degrees of freedom’) investigators face, will likely yield different results (53). This issue is particularly relevant given the ongoing concerns of reliability, reproducibility, and robustness in scientific research, including in traumatic brain injury literature (54). Unfortunately, researchers often fail to explicitly state which measure of ICV they have chosen for ROI normalization, or how ICV measures compare between groups, potentially impacting the reproducibility and reliability of findings across studies with similar designs. As we have illustrated here, this may be especially important in sports-related mTBI research where athletes may exhibit larger head sizes, and small sample sizes are common, often resulting in underpowered studies (55).

### 4.4 Limitations and future directions

This study had several limitations. On the one hand, we were fortunate to benefit from the recruitment of professional male Australian rules footballers: an inherently unique cohort who are traditionally difficult to recruit into neuroimaging studies given their demanding schedules. In many ways these athletes form an ideal cohort to study, as professional club doctors adhere to a standardized approach to diagnosing mTBI (27), which offers a consistent framework for examining post-concussion outcomes. However, given that these were professional athletes, there may be inherent cohort differences characteristic to these individuals (e.g., ICV) that make group comparison to a control cohort unmatched for this measure difficult. An ideal case–control study design would therefore include non-concussed control participants with similar athletic profiles (ideally from the same sporting code). Unfortunately, it is difficult to recruit such controls athletes, particularly those without any prior lifetime exposure to concussion (19). Other limitations of our study cohort included variable timing since injury, limited longitudinal data, and no information regarding concussion history prior to recruitment into the study. Indeed, it remains unclear whether the observed group differences are a signature of the acute and/or sub-acute injury, or instead reflect a long career as a professional athlete in which many concussive and sub-concussive impacts have likely been sustained (22, 23, 56). In addition, to maximise the number or participants included in the study, we included individuals scanned on two different MRI scanners. Although these were scanners of the same vendor with optimally matched acquisition protocols, there may be some scanner-related differences that contribute to our findings. However, our sensitivity analyses (see Supplementary material) provide support that our results are not primarily driven by scanner-related variability. Our cohort was also limited to male participants. Prior studies have reported sex-specific brain differences following concussion (57, 58), greater neurocognitive impairment and recovery times in females following concussion (59), and higher rates of concussion among professional female Australian rules footballers compared to professional males (60). Given the growing involvement of female participation in Australian rules football, we advocate for more inclusive and diverse cohorts in future concussion studies. Also, it is important to note that volumetric MRI, while sensitive to macrostructural brain alterations, is relatively insensitive to subtle microstructural injury following mTBI (61). Accordingly, the present volumetric findings likely reflect only a subset of underlying brain alterations, and we encourage readers to interpret them alongside prior multimodal evidence from this cohort (24–26). Moreover, while the present study focused on the hippocampus and amygdala, these regions are not uniquely vulnerable to concussion. Prior work has demonstrated that sports-related mTBI is associated with alterations in a distributed set of cortical and subcortical regions (13, 14), as well as widespread white matter tract abnormalities (61), and disruptions to large-scale brain networks (62, 63). More extensive whole-brain volumetric and multimodal imaging approaches will therefore be important in future studies to more comprehensively characterize the distributed, multi-scale effects of concussion. Finally, the use of the proportion method as a normalization approach introduces potential error into both the numerator (ROI estimate) and denominator (ICV estimate) (64, 65), and assumes ROIs scale proportionally with ICV, an assumption shown to depend on the ROI in question (65). We strongly encourage future studies with larger sample sizes to explore alternative approaches for ICV normalization that address these limitations and improve robustness.

In light of these limitations, particularly those concerning ROI normalization to ICV, we provide the following recommendations for future studies, several of which have been outlined in earlier work (52, 66): Firstly, we encourage researchers to increase analytical transparency by clearly stating the ICV estimation method chosen for volumetric normalization, and where possible, mitigating potential sources of bias by implementing quality control steps during analysis (52). Secondly, we recommend investigators report any group differences in ICV. If ICV estimates are indeed biased by brain volume (48), and unique intracranial volumes may be a distinct feature of elite sportspeople who compete in certain sports, especially when compared to non-athlete control cohorts, we believe this to be an important suggestion. Finally, if feasible, we recommend researchers consider exploring multiple analytical (or ‘forking’) paths, and report findings in the context of all possible decision points – an approach known as “multiverse analysis” (67–69). In the exploratory arm of this study, we provide a straightforward (albeit simple) example of multiverse analysis by focusing solely on FreeSurfer-derived estimates of ICV. Future studies could present how other analytical decisions previously shown to influence structural findings may also affect results, such as quality control steps (70–72), chosen analysis pipeline (including the software version) (73), and statistical frameworks (33, 65, 74).

Structural MRI studies hold meaningful promise for advancing our understanding of mTBI. These studies are increasingly utilized in sports-related concussion research, with quantitative approaches offering the potential to detect subtle post-injury brain changes, and make for potential biomarkers. This study provides evidence of possible structural brain differences in Australian rules footballers following sports-related mTBI, particularly the potential vulnerability of medial temporal lobe structures like the hippocampus. Large, longitudinal datasets will be valuable in assessing whether these subtle differences may be early signatures of long-term neurodegenerative changes, and whether quantifying these structural MRI differences in individuals may be a clinically valuable tool in the future. Importantly, we also demonstrate the importance of careful interpretation of clinical findings in the context of methodological variabilities, and advocate for future studies to explicitly state the intracranial volume estimation method used for volumetric normalization, report any group differences in intracranial volume, and explore multiple analytical approaches, ensuring that findings are considered within the context of all potential methodological decision points.

## Data Availability

The datasets presented in this article are not readily available because they include participant data that could compromise participant privacy. De-identified derived data that support the findings of this study are available upon reasonable request from the corresponding author. Requests to access the datasets should be directed to jackson.lee@student.unimelb.edu.au.
