# Supplementary material for: Structural brain differences in professional Australian rules footballers following mild traumatic brain injury: When head size matters
Source: Front Neurol. 2026 Jan 16;17:1701097. doi: 10.3389/fneur.2026.1701097 (PMC12855128; doi:10.3389/fneur.2026.1701097)
Supplement: Supplementary file 1 [file Supplementary_file_1.docx]

**Supplementary materials**

*The proportion method for ROI normalisation:*

Most commonly, correcting for the dependence of individual structure volumes on head size is achieved using either a proportion, residual, or analysis of covariance (ANCOVA) approach, for which the strengths, weaknesses, and underlying assumptions of each method have been previously outlined (1,2). In our case, there were significant group differences in ICV, which meant adjusting ROI volumes for ICV using the proportion method was the most appropriate choice, whereby:

ROI_adjusted_ = ROI_raw_ ÷ eTIV

This normalisation approach results in an adjusted ROI, which reflects the ratio between the ROI and eTIV for each subject (2). In this way, we were sensitive to *unconditional* group effects (the average volume difference for each ROI_adjusted_ between groups), and not *conditional* group effects as would be inferred from alternative normalisation approaches such as in analysis of covariance (ANCOVA) (1).

*Brief overview of ICV estimation techniques used for ROI normalisation:*

Structural MRI studies are common in the context of neurology, and increasingly common in research studies of concussion, where quantitative approaches are needed to identify potentially subtle changes that arise following injury (3–5). As brain volumes typically scale with head size, it is considered best practice to adjust volumes of interest for ICV when examining group-level differences (6,7), for which many approaches are available for ICV estimation. While manual tracing is considered the gold standard, it is often time consuming and impractical for large cohorts (8,9). Automated methods for ICV estimation are now widely accessible, and their application has become commonplace in neuroimaging research.

While FreeSurfer provides several ICV estimates across different pipelines, eTIV was chosen for ROI normalisation in the main arm of this project as it is arguably the most widely adopted estimation of ICV. eTIV is a registration-based estimate of ICV that is calculated from the alignment of the input MRI scan to the MNI305 brain atlas (10,11). To explore how the choice of ICV estimation method influenced volumetric findings, additional exploratory analyses were performed using two additional FreeSurfer-derived estimates. These additional ICV estimates were sbTIV (a segmentation-based estimate available through the SAMSEG (12) and SynthSeg (13) pipelines), and supratentorial volume (a surface-based volume estimation which FreeSurfer define as the volume of grey matter, white matter, brain stem, ventricles, choroid plexus, and vessels) (14). Although not technically an estimate of ICV, supratentorial volume was included in this study as it has been used to normalise hippocampal volumes in a previous sports-related mTBI study of high impact (15).

*On scanner harmonisation (including sensitivity analyses):*

Given that our cohort included participants scanned on two MRI scanners (albeit with optimally matched protocols), including participants from both scanners may introduce scanner-related bias into the study. Our main analyses were performed on all subjects to maximise sample size in this difficult to recruit cohort. Here, for transparency, we include several additional analyses to assess possible scanner-related effects:

1. Variance attributable to scanner

We quantified the proportion of variance in ICV normalised ROI volumes attributed to scanner using one-way ANOVA (see Supplementary Table 1). Scanner accounted for a small proportion of variance across regions ($\eta$² = 0.9-2.5%), with no significant scanner effects observed for hippocampal or amygdala volumes. Here, we also wish to highlight the near identical acquisition protocols used in this study across both scanners, with the only difference being the TE between scanners (2.5ms for Skyra, 2.6ms for Trio).

**Supplementary Table 1: Variance attributable to scanner.**

| *ROI*  *(eTIV normalised)* | *Statistic*  *(& p-value)* | $\eta$*² (% variance explained)* |
| --- | --- | --- |
| *Left hippocampus* | *F*(1, 66) = 1.38,  *p* = .244 | 0.020 (2.0%) |
| *Right hippocampus* | *F*(1, 66) = 0.80,  *p* = .374 | 0.012 (1.2%) |
| *Left amygdala* | *F*(1, 66) = 0.59,  *p* = .444 | 0.009 (0.9%) |
| *Right amygdala* | *F*(1, 66) = 1.72,  *p* = .194 | 0.025 (2.5%) |

1. Sensitivity analysis (Siemens Skyra only)

We ran a sensitivity analysis on our main volumetric (ROI) results by excluding participants scanned on the Siemens Trio (*n* = 12). In this analysis, the total sample size was *n* = 56 (29 controls, 27 mTBI). Our primary ROI findings in the subset of participants scanned on the Skyra were largely in line with our original results (see Supplementary Table 2), with the exception of the left amygdala, which no longer demonstrated a significant reduction in the mTBI group following Bonferroni correction.

**Supplementary Table 2: Sensitivity analysis.**

|  | Original finding reported in manuscript  (*n* = 68): | | Subjects scanned on Skyra only  (*n* = 56): | |
| --- | --- | --- | --- | --- |
| *ROI*  *(eTIV normalised)* | *Statistic*  *(& p-value)* | *Cohen’s d* | *Statistic*  *(& p-value)* | *Cohen’s d* |
| *Left hippocampus* | *t*(66) = 2.80  ***p* = .007** | 0.68 | *t*(54) = 2.39,  ***p* = .020** | 0.64 |
| *Right hippocampus* | *t*(66) = 4.00  ***p* = .0002** | 0.97 | *t*(54) = 3.30,  ***p* = .002** | 0.88 |
| *Left amygdala* | *t*(66) = 2.23  ***p* = .029** | 0.54 | *t*(54) = 1.44  *p* = .155 | 0.39 |
| *Right amygdala* | *t*(66) = 2.41  ***p* = .019** | 0.59 | *t*(54) = 2.47  ***p* = .017** | 0.66 |

Significant p-values are shown in bold.

1. ComBat harmonisation

ComBat harmonisation was performed on the entire output from FreeSurfer’s segmentation (*asegstats2table* output, excluding features with 0 values (*n* = 5)). Following ComBat harmonisation, and as in our original work, we normalised our ROIs of interest (hippocampal and amygdala volume) by the harmonised estimated total intracranial volume (eTIV) using the proportion method:

ROI_adjusted_ = ROI_ComBat-harmonised_ ÷ eTIV_ComBat-harmonised_

Supplementary Table 3 below shows our original ROI findings alongside results after ComBat harmonisation. We note here that the *t*-statistics and effect sizes (Cohen’s *d*) of group differences are nearly identical following ComBat to our originally reported findings.

**Supplementary Table 3: Volumetric results (mTBI vs controls) before and after ComBat harmonisation.**

|  | Without ComBat harmonisation  (original finding): | | With ComBat harmonisation: | |
| --- | --- | --- | --- | --- |
| *ROI*  *(eTIV normalised)* | *Statistic*  *(& p-value)* | *Cohen’s d* | *Statistic*  *(& p-value)* | *Cohen’s d* |
| *Left hippocampus* | *t*(66) = 2.80  ***p* = .007** | 0.68 | *t*(66) = 2.84,  ***p* = .006** | 0.68 |
| *Right hippocampus* | *t*(66) = 4.00  ***p* = .0002** | 0.97 | *t*(66) = 4.02,  ***p* = .0002** | 0.98 |
| *Left amygdala* | *t*(66) = 2.23  ***p* = .029** | 0.54 | *t*(66) = 2.22,  ***p* = .030** | 0.54 |
| *Right amygdala* | *t*(66) = 2.41  ***p* = .019** | 0.59 | *t*(66) = 2.47,  ***p* = .016** | 0.60 |

Significant p-values are shown in bold.

ComBat, like most harmonisation approaches, is primarily designed for multisite studies with larger sample sizes across sites (> 20-30 per site) to reliably estimate additive (shift) and multiplicative (scale), and has demonstrated poorer reliability at smaller sample sizes (< 20 for moving site, < 100 for reference site) (16–19). In this study, we unfortunately only had a small number of participants scanned on the Siemens Trio (*n* = 12; healthy controls: 8), meaning that Trio subjects’ data would likely be overfit, and any true biological differences present within the Trio group may be at risk of being removed during harmonisation. While we have included the ComBat harmonised findings here for transparency, we highlight that such an approach is likely not appropriate for small sample cohorts.

**Supplementary Table 4: Absolute ROI volumes.**

|  | *Left*  *hippocampus* | *Right*  *hippocampus* | *Left*  *amygdala* | *Right*  *amygdala* |
| --- | --- | --- | --- | --- |
| *mTBI* | 4775.32 (324.58) | 4952.70 (357.14) | 1942.20 (217.54) | 2115.74 (190.71) |
| *Acute mTBI* | 4775.49 (254.93) | 4908.37 (324.84) | 1906.61 (221.33) | 2067.44 (163.74) |
| *Sub-acute mTBI* | 4775.11 (403.90) | 5006.49 (398.50) | 1985.42 (212.72) | 2174.39 (210.18) |
| *Controls* | 4698.14 (551.75) | 4969.51 (512.27) | 1914.95 (258.75) | 2070.25 (254.14) |

All volumes have not been normalised for ICV, and are reported as means (standard deviation).

**Supplementary Table 5: Normalised ROI volumes between mTBI participants (subset) and controls.**

| *ROI* | *Group comparison* | *Mean difference (95% CI)* | *Statistic* | *Adjusted*  *p-value* | *Cohen’s d* |
| --- | --- | --- | --- | --- | --- |
| *Left hippocampus* | Control vs Acute | 0.013 (-0.003, 0.029) | *F*(2, 65) = 4.16  ***p* = .020** | .147 | 0.54 |
|  | Control vs Sub-acute | 0.019 (0.002, 0.037) |  | **.028** | 0.81 |
|  | Acute vs Sub-acute | 0.006 (-0.014, 0.026) |  | .736 | 0.30 |
| *Right hippocampus* | Control vs Acute | 0.026 (0.006, 0.039) | *F*(2, 65) = 7.87  ***p* < .001** | **.005** | 1.01 |
|  | Control vs Sub-acute | 0.026 (0.005, 0.040) |  | **.008** | 0.92 |
|  | Acute vs Sub-acute | -4.04 × 10^-5^ (0.020, -0.020) |  | .999 | 0.00 |
| *Left amygdala* | Control vs Acute | 0.007 (-0.001, 0.016) | *F*(2, 65) = 2.52  *p* = .088 | **-** | **-** |
|  | Control vs Sub-acute | 0.006 (-0.004, 0.015) |  | **-** | **-** |
|  | Acute vs Sub-acute | -0.002 (-0.012, 0.009) |  | **-** | **-** |
| *Right amygdala* | Control vs Acute | 0.008 (0.000, 0.015) | *F*(2, 65) = 3.21  ***p* = .047** | **.045** | 0.70 |
|  | Control vs Sub-acute | 0.005 (-0.004, 0.013) |  | .372 | 0.44 |
|  | Acute vs Sub-acute | -0.003 (-0.013, 0.006) |  | .698 | 0.30 |

For each pairwise comparison, the mean difference (with 95% CI) are shown as the proportional difference between groups for each ROI. F-statistics and one-way ANOVAs (with uncorrected *p*-values reported) are given. Significant p-values (adjusted after Tukey HSD tests) are shown in bold.

|  | *eTIV* | *sbTIV* | *Supratentorial volume* |
| --- | --- | --- | --- |
| *mTBI* | 1779.00 (141.79) | 1765.22 (128.41) | 1201.30 (99.73) |
| *Acute* | 1761.55 (133.53) | 1746.37 (106.82) | 1191.36 (93.54) |
| *Sub-acute* | 1800.18 (153.52) | 1788.11 (151.58) | 1213.37 (109.06) |
| *Controls* | 1648.84 (150.18) | 1701.91 (145.10) | 1145.29 (102.11) |
| *Statistic,*  *p-value* | *t*(66) = 3.65,  ***p* = .0005**  *F*(2, 65) = 6.88,  ***p* = .002** | *t*(66) = 1.89,  *p* = .063  *F*(2, 65) = 2.12,  *p* = .128 | *t*(66) = 2.28,  ***p* = .026**  *F*(2, 65) = 2.75,  *p* = .072 |

**Supplementary Table 6: Intracranial volume estimates.**

Reported as means (standard deviation) (cm^3^). Significant p-values are shown in bold. Note: the eTIV column (shaded in grey) represent the results reported in Table 1. *F*-statistics reflect group-level comparisons between controls and subset mTBI cohorts, while *t*-statistics reflect comparisons between controls and the whole mTBI cohort.

| *ROI* | *Group comparison* | *eTIV* | | *sbTIV* | | *Supratentorial volume* | | | |
| --- | --- | --- | --- | --- | --- | --- | --- | --- | --- |
|  |  | *Statistic* | *Adjusted p-value* | *Statistic* | *Adjusted p-value* | | *Statistic* | | *Adjusted p-value* |
| *Left hippocampus* | Control vs Acute | *F*(2, 65) = 4.16 | .147 | *F*(2, 65)  = 0.78 | .936 | *F*(2, 65) = 1.25 | | .693 | |
|  | Control vs Sub-acute |  | **.028** |  | .438 |  |  | .282 | |
|  | Acute vs Sub-acute |  | .736 |  | .700 |  |  | .782 | |
| *Right hippocampus* | Control vs Acute | *F*(2, 65) = 7.87 | **.005** | *F*(2, 65)  = 2.42 | .176 | *F*(2, 65) = 3.45 | | .067 | |
|  | Control vs Sub-acute |  | **.008** |  | .202 |  |  | .138 | |
|  | Acute vs Sub-acute |  | .999 |  | .999 |  |  | .986 | |
| *Left amygdala* | Control vs Acute | *F*(2, 65) = 2.52 | **.**109 | *F*(2, 65)  = 0.44 | .619 | *F*(2, 65) = 0.89 | | .393 | |
|  | Control vs Sub-acute |  | .305 |  | .936 |  |  | .825 | |
|  | Acute vs Sub-acute |  | .927 |  | .890 |  |  | .847 | |
| *Right amygdala* | Control vs Acute | *F*(2, 65) = 3.21 | **.045** | *F*(2, 65)  = 0.62 | .546 | *F*(2, 65) = 1.45 | | .217 | |
|  | Control vs Sub-acute |  | .372 |  | .999 |  |  | .958 | |
|  | Acute vs Sub-acute |  | .698 |  | .646 |  |  | .501 | |

**Supplementary Table 7: Analysis of relative ROI volumes with different FreeSurfer ICV estimations.**

Relevant group-level statistical results (subset mTBI cohort vs controls) are shown for each ROI after normalisation with either eTIV, sbTIV, or supratentorial volume. Significant uncorrected *p*-values (adjusted after Tukey HSD tests) are shown in bold. Note: the eTIV columns (shaded in grey) represent the results reported in Supplementary Figure 1.

**
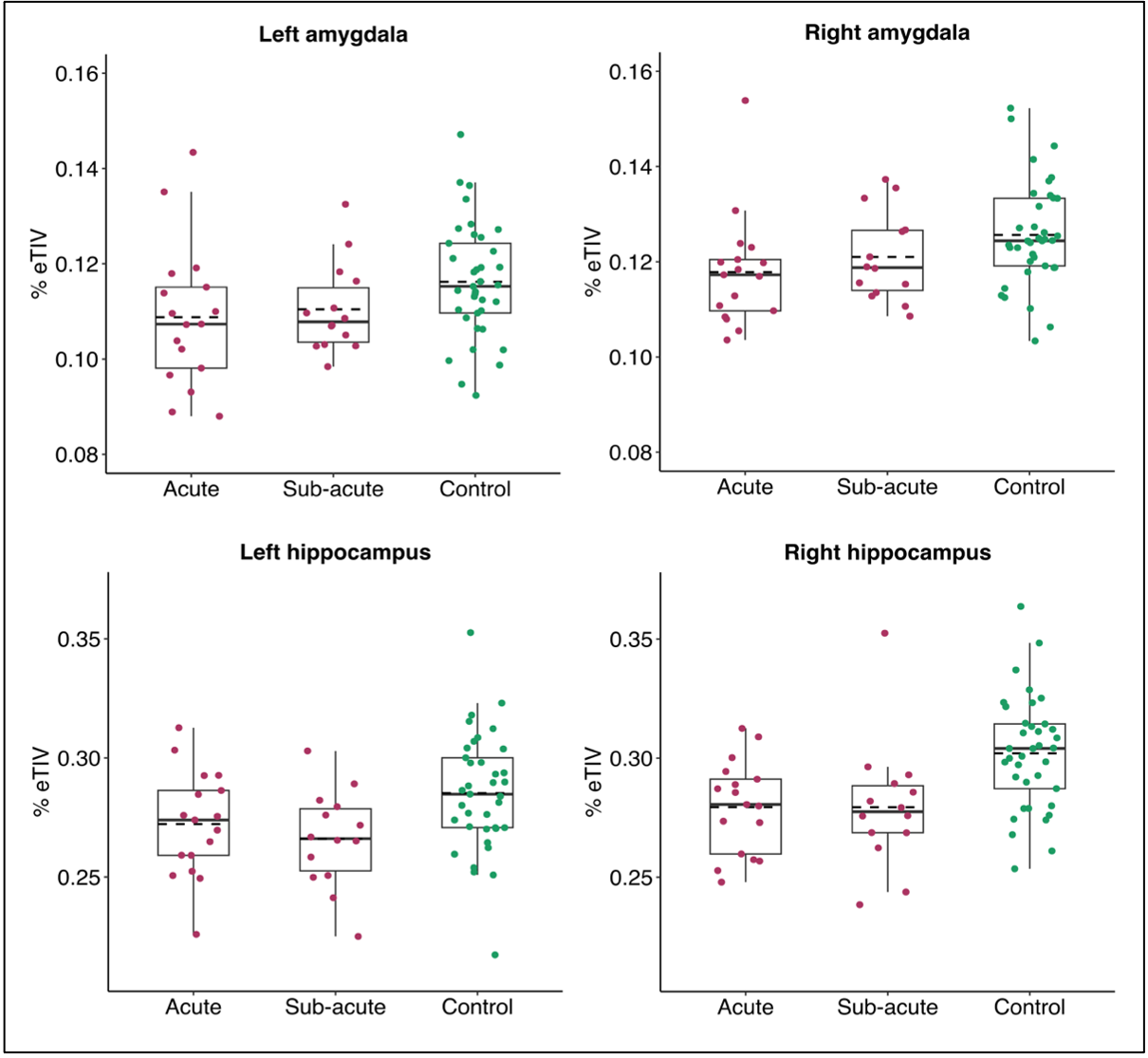
**

**Supplementary Figure 1: Normalised ROI volumes for mTBI participants (subset) and controls.** Box plots depicting normalised ROI volumes. The interquartile range is represented by each box (median indicated by a solid horizontal line, mean indicated by a dotted horizontal line). Whiskers extend to the minimum and maximum values within 1.5 times the IQR.

**
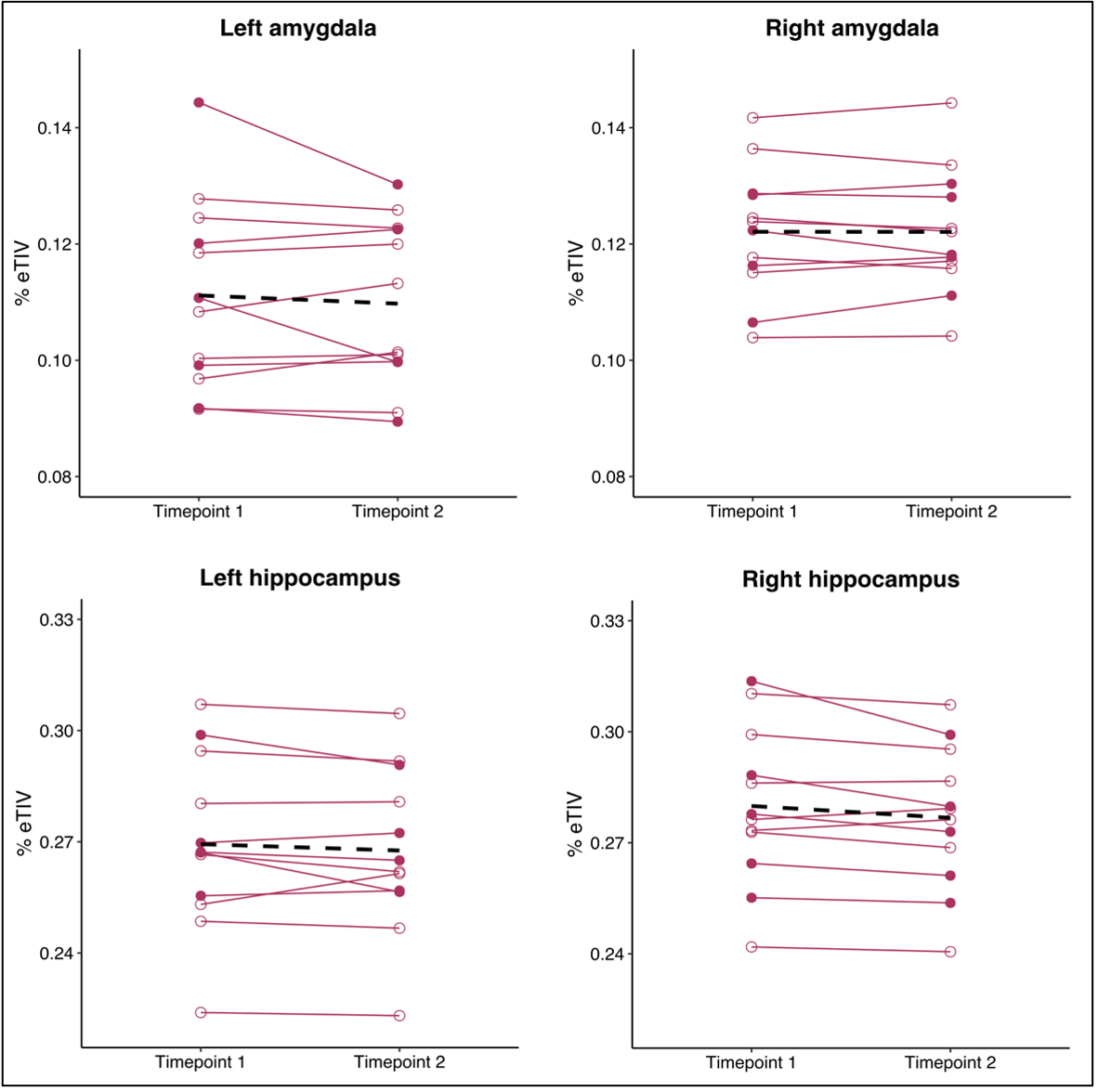
**

**Supplementary Figure 2: Longitudinal change in ROI volumes across timepoints.** Line graph depicting change in ROI volume (as a % of eTIV) between first and follow-up scan in Australian rules footballers following mTBI. Average volume change between timepoints is shown by the dashed black line. Line colours depict each mTBI cohort: acute mTBI (*n* = 5) shown as filled circles, and sub-acute mTBI (n = 7) shown as open circles.

**
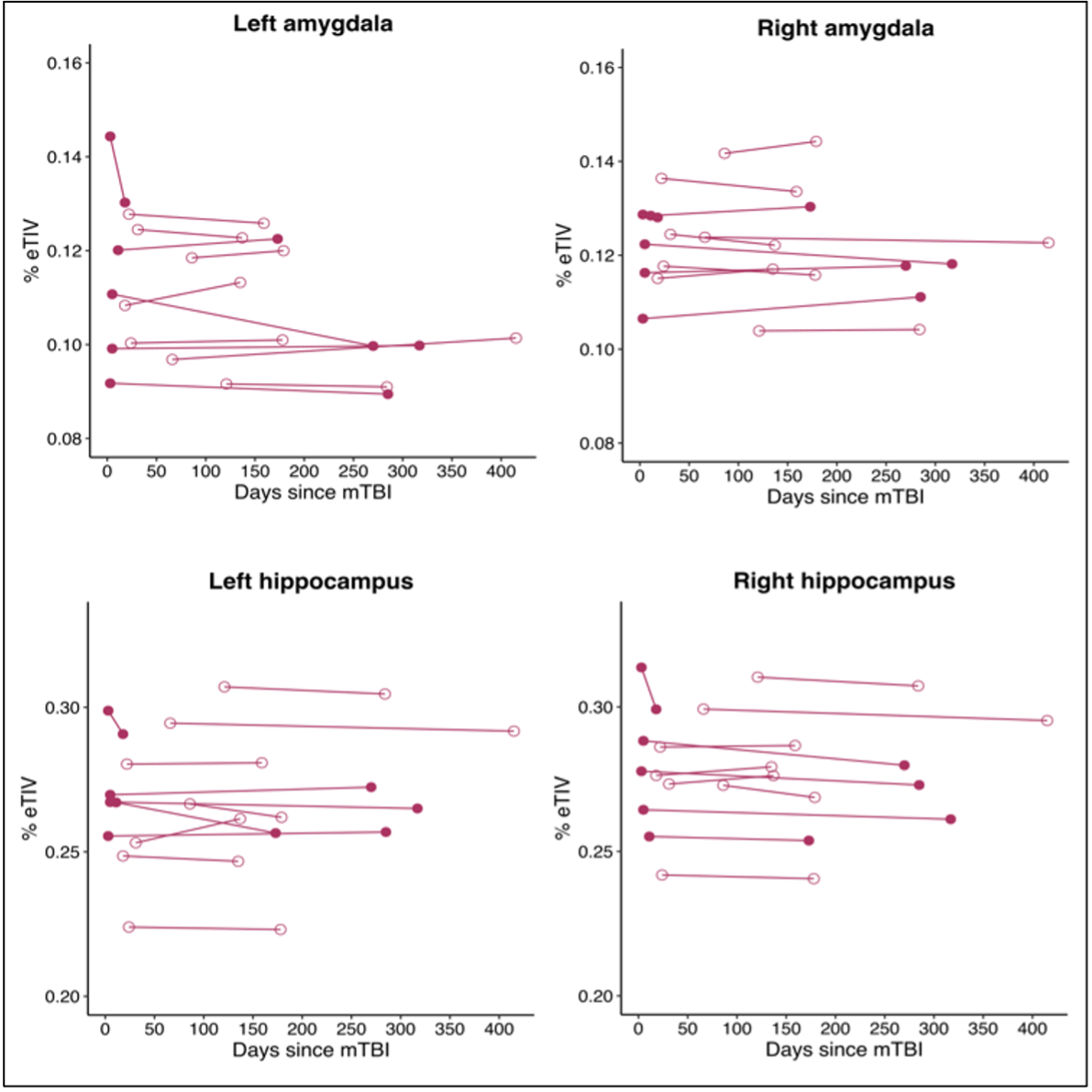
**

**Supplementary Figure 3: Longitudinal change in ROI volumes relative to days since injury.** Line graph depicting change in ROI volume (as a % of eTIV) over time (number of days since concussion). Line colours depict each mTBI cohort: acute mTBI (*n* = 5) shown as filled circles, and sub-acute mTBI (n = 7) shown as open circles.

**
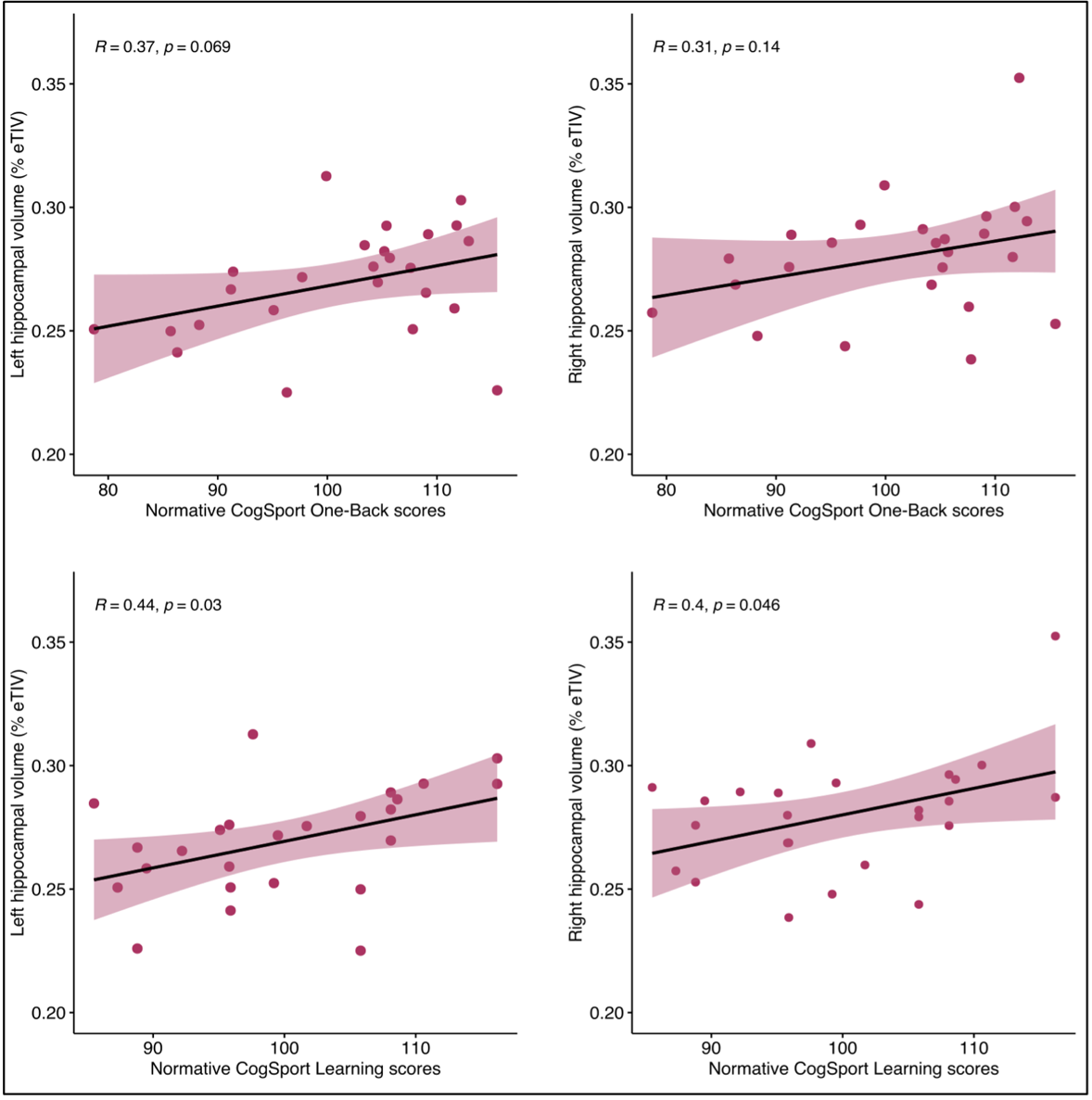
**

**Supplementary Figure 4: Correlation between hippocampal volume and CogSport cognitive measures.** Scatterplots depicting correlation between mTBI participants (left and right) hippocampal volume (shown as a % of eICV) and cognitive scores from the one-back and learning tests shown as normative scores, where 100 reflects mean score from healthy individuals.

**
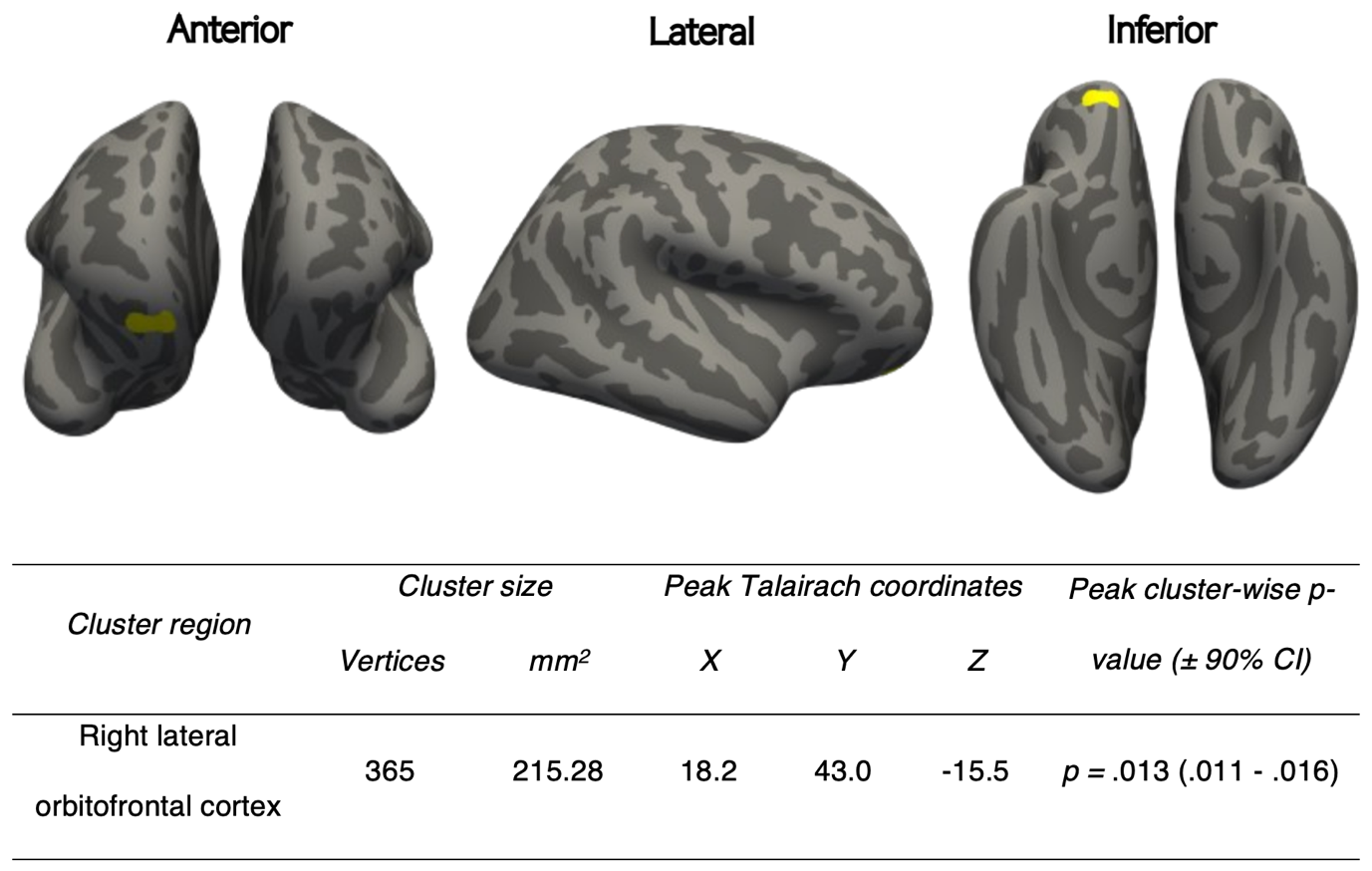
**

| *Cluster region* | *Cluster size* | | *Peak Talaraich coordinates* | | | *Peak cluster-wise p-value (*$\pm$ *90% CI)* |
| --- | --- | --- | --- | --- | --- | --- |
|  | *Vertices* | *mm^2^* | *X* | *Y* | *Z* |  |
| *Right lateral orbitofrontal cortex* | 365 | 215.28 | 18.2 | 43.0 | -15.5 | *p =* .013 (.011 - .016) |

**Supplementary Figure 5: Significant cluster (FreeSurfer output).** The right hemisphere is shown from anterior, lateral, and inferior views, with a significant cluster in yellow indicating reduced thickness in Australian rules footballers compared to controls at the right lateral orbitofrontal cortex (3D rendering created using FreeView). Accompanying the renders is a table summarising the significant cluster information as output from FreeSurfer.

**
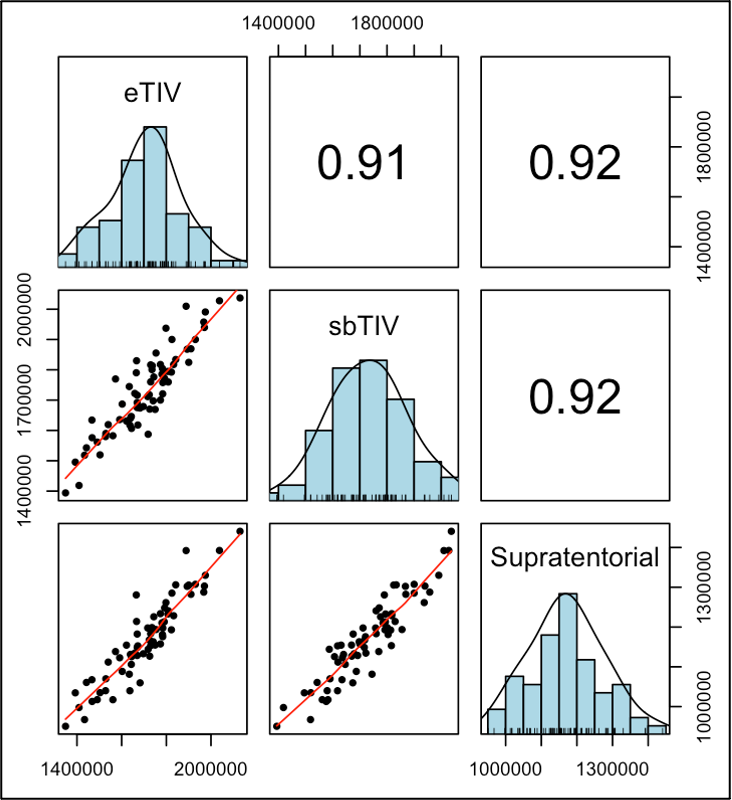
**

**Supplementary Figure 6: Correlations between each FreeSurfer ICV estimation method.** The main diagonal displays the distribution of each ICV estimate, while the lower diagonal depicts scatter plots for each pair of ICV estimates. The upper diagonal shows Pearson correlation coefficients, all of which were statistically significant (*p* < .001).

**References**

1. O’Brien LM, Ziegler DA, Deutsch CK, Kennedy DN, Goldstein JM, Seidman LJ, et al. Adjustment for whole brain and cranial size in volumetric brain studies: a review of common adjustment factors and statistical methods. Harvard Review of Psychiatry. 2006 June;14(3):141.

2. O’Brien LM, Ziegler DA, Deutsch CK, Frazier JA, Herbert MR, Locascio JJ. Statistical adjustments for brain size in volumetric neuroimaging studies: some practical implications in methods. Psychiatry Res. 2011 Aug 30;193(2):113–22.

3. Eierud C, Craddock RC, Fletcher S, Aulakh M, King-Casas B, Kuehl D, et al. Neuroimaging after mild traumatic brain injury: review and meta-analysis. Neuroimage Clin. 2014 Jan 4;4:283–94.

4. Bigler ED. Structural neuroimaging in sport-related concussion. Int J Psychophysiol. 2018 Oct;132(Pt A):105–23.

5. Bigler ED. Volumetric MRI findings in mild traumatic brain injury (mTBI) and neuropsychological outcome. Neuropsychol Rev. 2023 Mar 1;33(1):5–41.

6. Backhausen LL, Herting MM, Tamnes CK, Vetter NC. Best practices in structural neuroimaging of neurodevelopmental disorders. Neuropsychol Rev. 2022 June 1;32(2):400–18.

7. Barnes J, Ridgway GR, Bartlett J, Henley SMD, Lehmann M, Hobbs N, et al. Head size, age and gender adjustment in MRI studies: a necessary nuisance? NeuroImage. 2010 Dec 1;53(4):1244–55.

8. Klasson N, Olsson E, Rudemo M, Eckerström C, Malmgren H, Wallin A. Valid and efficient manual estimates of intracranial volume from magnetic resonance images. BMC Med Imaging. 2015 Feb 18;15(1):5.

9. Nordenskjöld R, Malmberg F, Larsson EM, Simmons A, Brooks SJ, Lind L, et al. Intracranial volume estimated with commonly used methods could introduce bias in studies including brain volume measurements. Neuroimage. 2013 Dec;83:355–60.

10. Buckner RL, Head D, Parker J, Fotenos AF, Marcus D, Morris JC, et al. A unified approach for morphometric and functional data analysis in young, old, and demented adults using automated atlas-based head size normalization: reliability and validation against manual measurement of total intracranial volume. NeuroImage. 2004 Oct 1;23(2):724–38.

11. Nerland S, Stokkan TS, Jørgensen KN, Wortinger LA, Richard G, Beck D, et al. A comparison of intracranial volume estimation methods and their cross‐sectional and longitudinal associations with age. Hum Brain Mapp. 2022 June 16;43(15):4620–39.

12. Puonti O, Iglesias JE, Van Leemput K. Fast and sequence-adaptive whole-brain segmentation using parametric Bayesian modeling. NeuroImage. 2016 Dec 1;143:235–49.

13. Billot B, Greve DN, Puonti O, Thielscher A, Van Leemput K, Fischl B, et al. SynthSeg: segmentation of brain MRI scans of any contrast and resolution without retraining. Med Image Anal. 2023 May;86:102789.

14. Fischl B, Salat DH, Busa E, Albert M, Dieterich M, Haselgrove C, et al. Whole brain segmentation: automated labeling of neuroanatomical structures in the human brain. Neuron. 2002 Jan 31;33(3):341–55.

15. Singh R, Meier TB, Kuplicki R, Savitz J, Mukai I, Cavanagh LM, et al. Relationship of collegiate football experience and concussion with hippocampal volume and cognitive outcomes. JAMA. 2014 May 14;311(18):1883–8.

16. Fortin JP, Cullen N, Sheline YI, Taylor WD, Aselcioglu I, Cook PA, et al. Harmonization of cortical thickness measurements across scanners and sites. NeuroImage. 2018 Feb 15;167:104–20.

17. Orlhac F, Eertink JJ, Cottereau AS, Zijlstra JM, Thieblemont C, Meignan M, et al. A guide to ComBat harmonization of imaging biomarkers in multicenter studies. Journal of Nuclear Medicine. 2022 Feb 1;63(2):172–9.

18. Kim ME, Gao C, Cai LY, Yang Q, Newlin NR, Ramadass K, et al. Empirical assessment of the assumptions of ComBat with diffusion tensor imaging. JMI. 2024 Apr;11(2):024011.

19. Jodoin PM, Edde M, Girard G, Dumais F, Theaud G, Dumont M, et al. Challenges and best practices when using ComBAT to harmonize diffusion MRI data. Sci Rep. 2025 Nov 24;15(1):41508.
